# Motor imagery therapy improved upper limb motor function in stroke patients with hemiplegia by increasing functional connectivity of sensorimotor and cognitive networks

**DOI:** 10.3389/fnhum.2024.1295859

**Published:** 2024-02-19

**Authors:** Wan Liu, Xinxin Cheng, Jiang Rao, Jiawen Yu, Zhiqiang Lin, Yao Wang, Lulu Wang, Danhui Li, Li Liu, Run Gao

**Affiliations:** ^1^Department of Rehabilitation, The Affiliated Brain Hospital of Nanjing Medical University, Nanjing, China; ^2^Department of Rehabilitation, Changzhou Ruihong Hospital, Changzhou, China; ^3^Graduate Department, Nanjing Sports Institute, Nanjing, China

**Keywords:** stroke, motor imagery therapy, upper limb motor function, sensorimotor network, functional connectivity

## Abstract

**Background:**

Motor imagery therapy (MIT) showed positive effects on upper limbs motor function. However, the mechanism by which MIT improves upper limb motor function is not fully understood. Therefore, our purpose was to investigate the changes in functional connectivity (FC) within and outside the sensorimotor network (SMN) induced by MIT associated with improvement in upper limb motor function in stroke patients.

**Methods:**

A total of 26 hemiplegic stroke patients were randomly divided into MIT (*n* = 13) and control (*n* = 13) groups. Fugl-Meyer Assessment Upper Extremity Scale (FMA-UL), Modified Barthel Index (MBI) and resting-state functional magnetic resonance imaging (rs-fMRI) were evaluated in the two groups before treatment and 4 weeks after treatment. The efficacy of MIT on motor function improvement in stroke patients with hemiplegia was evaluated by comparing the FMA-UL and MBI scores before and after treatment in the two groups. Furthermore, the FC within the SMN and between the SMN and the whole brain was measured and compared before and after different treatment methods in stroke patients. The correlation analysis between the improvement of upper limbs motor function and changes in FC within the SMN and between the SMN and the whole brain was examined.

**Results:**

The FCs between ipsilesional primary motor cortex (M1.I) and contralateral supplementary motor area (SMA.C), M1.I and ipsilesional SMA (SMA.I), and SMA.C and contralateral dorsolateral premotor cortex (DLPM.C) significantly increased in the control group but decreased in the MIT group; while the FC between SMA.C and contralateral primary somatosensory cortex (S1.C) significantly increased in the control group but showed no significant difference in the MIT group. The FCs between M1.I and the ipsilesional hippocampal gyrus and ipsilesional middle frontal gyrus significantly decreased in the control group but increased in the MIT group; while the FC in the contralateral anterior cingulate cortex significantly increased in the MIT group but there was no significant difference in the control group. The results of the correlation analysis showed that the differences in abnormal intra-FCs within the SMN negatively correlated with the differences in FMA and MBI, and the difference in abnormal inter-FCs of the SMN positively correlated with the differences in FMA and MBI.

**Conclusions:**

MIT can improve upper limb motor function and daily activities of stroke patients, and the improvement effect of conventional rehabilitation therapy (CRT) combined with MIT is significantly higher than that of CRT alone. CRT may improve the upper limb motor function of stroke patients with hemiplegia mainly through the functional reorganization between SMN, while MIT may mainly increase the interaction between SMN and other brain networks.

## 1 Introduction

Stroke is one of the leading causes of death and disability worldwide (Chien et al., 2020), dramatically affecting the independence and quality of life of survivors. After stroke, about 80% of the patients continue to have upper extremity motor impairment (Zhuang et al., 2021). Stroke patients with upper extremity functional limitation are particularly susceptible to problems in performing daily activities (Ikbali Afsar et al., 2018). Currently, several advanced treatment techniques have been developed and used for stroke survivors. Nevertheless, we face several challenges in rehabilitation after stroke. In clinical practice, the recovery of upper limb and hand function after stroke is difficult.

Motor imagery training (MIT) is widely used in the rehabilitation of upper limb motor function after stroke as an active central intervention technology (Villa-Berges et al., 2023). MIT has no special requirements for the level of motor function of patients. Patients can improve their motor performance by repeatedly imagining and simulating the specified actions (Wang et al., 2019). These studies observed that motor imagery, as a complementary technique, improved upper limbs motor function (Nam et al., 2019; Vourvopoulos et al., 2019; Barclay et al., 2020; Gaughan and Boe, 2021; Hilt et al., 2023). However, there is no consensus regarding the intervention protocol for the application of motor imagery. Therefore, we propose a protocol of embodied motor imagery on the basis of motor imagery. The theoretical basis of embodied motor imagery is the modern cognitive concept, that is, the embodied cognition. The modern concept of embodied cognition is different from the traditional concept of cognition. The modern concept of embodied cognition believes that the brain, body, and environment interact and dynamically couple into a cognitive system (Ziemke, 2016). Also, some of the studies, which used multisensory supports found improvements in motor imagery, as these cues facilitate sensory integration (Cho et al., 2013; Rayegani et al., 2014; Oostra et al., 2015; Kumar et al., 2016). More importantly, further understanding of the underlying mechanism of MIT can help the therapist optimize the treatment plan and improve the efficiency of rehabilitation.

With the rapid development of neuroimaging, our understanding of MIT in stroke rehabilitation has made substantial progress. Early functional imaging studies have shown that the primary motor cortex (M1), premotor cortex (PMC), and supplementary motor area (SMA) are activated during motor imagery tasks and motor execution (Lotze et al., 1999; Johnson et al., 2002). Subsequent task-state functional magnetic resonance studies showed that compared to the control group, the number of activated voxels in the contralateral somatosensory motor cortex (SMC) of the MIT group increased, and the number of activated voxels in the contralateral SMC was positively correlated with hand function scores (Liu et al., 2014). A Granger causality analysis showed that there were tighter connections in the cortical motor network in the stroke patients than in the controls during motor imagery, and the patients showed more effective connectivity in the intact hemisphere (Wang et al., 2016). Similarly, recent resting-state functional magnetic resonance (rs-fMRI) studies concluded that the MIT group showed increased functional connectivity (FC) of the ipsilesional M1 with the ipsilesional precentral and postcentral gyri, middle cingulate gyrus, and supramarginal gyrus after treatment, while the control group showed decreased FC between these regions (Wang et al., 2019). In addition to the sensorimotor network (SMN), a review combining the data from 75 papers revealed that MIT consistently recruits a large fronto-parietal network (Hetu et al., 2013). A recent rs-fMRI study showed that the slow-5 fractional amplitude of low-frequency fluctuation (fALFF) value of the inferior parietal lobule (IPL) in the MIT group was higher than that in the control group. The FCs of the affected IPL in the MIT group also showed significant changes. This change is related to the improvement in Fugl-Meyer Assessment Upper Extremity Scale (FMA-UL) (Wang et al., 2020). In summary, most of the previous functional imaging studies have shown that MIT activates motor-related brain regions, or causes increased FC between motor-related brain regions and other brain regions. In recent years, rs-fMRI studies have found that MIT not only changes the activity of motor-related brain regions, but also changes the activity of brain regions related to memory, execution, etc., such as middle cingulate gyrus and fronto-parietal network. However, none of the previous studies has fully revealed the neural network mechanism behind MIT from the perspective of FC within the SMN and between the SMN and other network brain regions. Our study plans to comprehensively and systematically explore the mechanism of MIT from this perspective.

Therefore, we randomly divided the study subjects into MIT and control groups. The control group received traditional rehabilitation training (CRT), while the MIT group received embodied MIT in addition to CRT. FMA-UL, Modified Barthel Index (MBI) and rs-fMRI were evaluated in the two groups before treatment and after treatment. The efficacy of embodied MIT on motor function improvement in stroke patients with hemiplegia was evaluated by comparing the FMA-UL and MBI scores before and after treatment in the two groups. Rs-fMRI was performed to investigate the changes in FC within the SMN and the connection between the SMN and other brain networks induced by MIT. We speculated that MIT improves the upper limb motor function in stroke patients not only through functional reorganization within the SMN, but also between SMN and other brain networks.

## 2 Materials and methods

### 2.1 Participants

This study was a randomized controlled study, and the evaluators were blinded. A total of 26 first-time stroke patients were enrolled, including 20 patients with cerebral infarction and 6 patients with cerebral hemorrhage. All patients were from the Department of Rehabilitation Medicine, the Affiliated Brain Hospital of the Nanjing Medical University. The inclusion criteria for the study participants were: (1) diagnosed by computed tomography or MRI as first-ever subcortical ischemic or hemorrhagic stroke; (2) 3–12 months after stroke onset; (3) unilateral limb hemiplegia (Modified Brunnstrom classification as grade I–IV); (4) Mini-mental State Examination (MMSE) ≥27; (5) Kinesthetic and Visual Imagery Questionnaire (KVIQ-10) ≥25; (6)18–75 years old; (7) right-handedness. The exclusion criteria were: (1) severe spasticity of affected upper limbs (modified Ashworth spasticity scale > 2); (2) aphasia, disturbance of consciousness, severe hearing and visual impairment; (3) significant pain in affected side (Ten-point Visual Analog Scale > 4); (4) severe primary heart, lung, liver, kidney or hematopoietic system diseases; (5) after craniectomy or cranioplasty. (6) MRI contraindications. After baseline clinical assessment, a therapist divided the eligible patients into MIT and control groups using the random number table, with 13 patients in each group. A total of 6 participants were excluded due to excessive head motion (cumulative translation or rotation ≥3.0 mm). As a result, the study included 20 patients (8 in the MIT group and 12 in the control group).

This study was granted approval by the responsible Human Participants Ethics Committee of the Affiliated Brain Hospital of the Nanjing Medical University (Nos.2017-KY038), located in Nanjing, China. All participants signed a written informed consent prior to participation.

### 2.2 Clinical intervention

All patients received CRT 2 h per day, 5 days per week for 4 weeks and conventional medication. CRT included physical therapy, occupational therapy, electrical stimulation, and Chinese acupuncture. Physical therapy and occupational therapy involve joint activity training, muscle strength training, fine motor training, activities of daily living training and task-related gross motor and dexterity exercises. Besides this, the MIT group received 30 min of specific embodied motion imagery training. Embodied MIT involves motor schema training, perceptual integration training, and body intentionality training. The focus is on mirror motion imitation and enhancement of sensory-motor experience, and the establishment of procedural and memorization of action patterns. Detailed information regarding embodied MIT is included in the Supplementary material.

### 2.3 Clinical scales assessment and imaging data acquisition

All patients were assessed using MMSE and KVIQ-10 at admission. Before and after the clinical intervention, each patient received an assessment of upper extremity motor function using the FMA_UL scale and an assessment of independence in daily activities using MBI by a physician who was blinded to the treatment condition. Each patient underwent rs-fMRI before and after clinical intervention. The detailed parameters of rs-fMRI acquisition of the patients are summarized in the Supplementary material.

### 2.4 Image preprocessing

Image preprocessing was performed utilizing Data Processing Assistant for Resting-State fMRI (DPARSF 4.4, http://www.restfmri.net) based on Matlab2013b platform (Yan et al., 2016). The images from the patients with right lesions were flipped. The first 10 volumes of functional images were removed for each patient. Then, the remaining 241 images were corrected using slice-timing and realignment, accounting for head motion, normalized to standard space using DARTEL, resampled to a 3 × 3 × 3 mm3 voxel size, regress nuisance variable, and spatially smoothed with 4 mm full width at half maximum (FWHM). The nuisance variables include 24 motion parameters (six head motion parameters, six head motion parameters one time point before, and the 12 corresponding squared items), a global signal, a white matter signal, and a cerebrospinal fluid signal. Finally, a temporal filter (0.01–0.08 Hz) was applied to reduce low-frequency drift and high-frequency physiological noise. In addition, patients with excessive head motion (cumulative translation or rotation ≥3.0 mm) were excluded.

### 2.5 Seed selection and FC analysis

For regions of interest (ROI)-wise connectivity analysis, we selected 14 subcortical and cortical sensory- and motor-related brain regions based on previous studies. The regions included the bilateral M1, bilateral SMA, bilateral primary somatosensory cortex (S1), bilateral secondary somatosensory cortex (S2), bilateral basal ganglia (BG), bilateral dorsolateral premotor cortex (dlPM), and bilateral ventrolateral premotor cortex (vlPM) (Min et al., 2015). The detailed positions of the 14 ROIs of the sensorimotor network (spheres of 5 mm radius) are provided in the Supplementary Table S1.

ROI-wise connectivity was measured by extracting the mean time seriers from each ROI listed above and calculating the Pearson's correlation coefficients between each ROI pair. The resulting correlation coefficients were then Fisher-transformed into Z-scores to increase normality.

To further investigate the FC of the SMN and the whole brain, we calculated seed-based whole-brain voxel-wise FC. The ipsilesional M1 (left side) was selected as the seed because it is the most prominent brain area of functional reorganization following MIT (Zhang et al., 2016; Wang et al., 2019). The whole-brain resting-state FC maps were obtained for the left M1 in stroke patients using the DPARSF toolbox. Similarly, the correlation coefficients of each voxel were normalized to Z-scores using the Fisher's r-to-z transformation.

### 2.6 Statistical analyses

We used the SPSS (Statistical Product and Service Solutions) v 24.0 software for statistical analysis. The age, months after stroke onset, KVIQ score, and lesion volume between the MIT and control groups were compared using independent sample t-test, while gender, stroke type, and side of lesion were compared using the Chi-square test. A p-value of <0.05 indicated statistically significant differences. Then, we performed the repeated-measures analysis of variance (ANOVA) on FMA_UL and MBI to verify the efficiency of MIT, taking group (2 levels: MIT and control) as the between-subject factor and time (2 levels: before and after intervention) as the within-subject factor. *Post hoc* analysis was conducted by performing the *t*-test to compare FMA_UL and MBI between the 2 groups before and after the intervention.

For imaging data, analyses of the ROI-wise connectivity were performed using repeated-measures analysis of covariance (ANCOVA) and *t*-test *post hoc* (False Discovery Rate (FDR) corrected, *P* < 0.05). We used demographic data (age, gender) and clinical data (months after stroke onset, lesion volume) as covariables. Then, using FMRIB Software Library (FSL) Randomize (www.fmrib.ox.ac.uk/fsl/randomise), voxel wise 2 × 2 (group × time) repeated ANCOVA was carried out to investigate the brain regions with different alterations in FC maps (seed-based whole-brain voxel-wise connectivity) between the 2 groups. We set significance with a threshold-free cluster enhancement family-wise error rate (TFCE-FWE) corrected cluster *P* < 0.05 and the cluster size > 10 voxels (270 mm3), and used demographic data (age, gender) and clinical data (months after stroke onset, lesion volume) as covariables. The FC values from the clusters with significant time × group interaction were extracted and averaged for *post hoc* analysis, in which paired *t*-tests were used to compare before and after intervention in each group.

Finally, signals were extracted from the significant clusters between after treatment and before treatment (ΔFCs). Partial correlation analyses were carried out to reveal relationships between ΔFCs and ΔFMA_UL (difference between after treatment and before treatment) or ΔMBI (difference between after treatment and before treatment) after adjusting for the effects of age, gender, months after stroke onset, and lesion volume (Bonferroni-corrected, *P* < 0.05).

## 3 Results

### 3.1 Demographic and clinical characteristics

Detailed demographic and clinical data for the patients are shown in Table 1. We found no significant differences in age, gender, months after stroke onset, stroke type, side of lesion, lesion volume, and KVIQ score between the MIT and control groups (all *P* > 0.05) (Table 1). However, significant group × time interactions were observed in FMA_UL (*F* = 108.32, *P* < 0.001) and MBI (*F* = 15.86, *P* = 0.001). What's more, FMA_UL and MBI scores before the intervention were comparable between the 2 groups (all *P* > 0.05) (Table 1). After intervention, the scores in the MIT group were significantly higher than those in the control group (all *P* < 0.05) (Table 1).

**Table 1:** Demographic and clinical information for the participants.

	**MIT (*n =* 8)**	**Control (*n =* 12)**	***T/χ^2^***	***P***
Age in years, M ± SD	58.63 ± 11.89	60.17 ± 11.87	−0.284 (*T*)	0.779
Gender, male/female, *n*	4/4	7/5	0.135 (*χ^2^*)	0.714
Months after stroke onset, M ± SD	4.13 ± 0.99	4.33 ± 2.02	−0.270 (*T*)	0.790
Stroke type, ischemia/hemorrhage, *n*	5/3	7/5	0.035 (*χ^2^*)	0.852
Side of lesion, left/right, *n*	4/4	7/5	0.135 (*χ^2^*)	0.714
Lesion volume, cm3, M ± SD	3.71 ± 4.30	3.77 ± 3.61	−0.031 (*T*)	0.976
KVIQ, M ± SD	31.88 ± 4.58	31.25 ± 4.33	0.309 (*T*)	0.761
MBI before intervention, M ± SD	59.75 ± 4.30	60.17 ± 3.79	−0.229 (*T*)	0.822
MBI after intervention, M ± SD	77.75 ± 4.03	73.50 ± 3.83	2.384 (*T*)	0.028
FMA_UL before intervention, M ± SD	17.13 ± 2.23	17.42 ± 2.35	−0.277 (*T*)	0.785
FMA_UL after intervention, M ± SD	32.63 ± 3.25	23.67 ± 3.28	6.001 (*T*)	< 0.001

Age, months after stroke onset, KVIQ score and lesion volume between the two groups were compared by independent sample t-test, while gender, stroke type and side of lesion were tested by chi-square test. FMA_UL and MBI were performed using the repeated-measures analysis of variance and t tsets post hot. MIT, motor imagery training; KVIQ, Kinesthetic and Visual Imagery Questionnaire; MBI, Modified Barthel Index; FMA_UL, Fugl-Meyer Assessment Upper Limb subscale.

### 3.2 ROI-wise connectivity within the sensorimotor network

**Figure 1:**
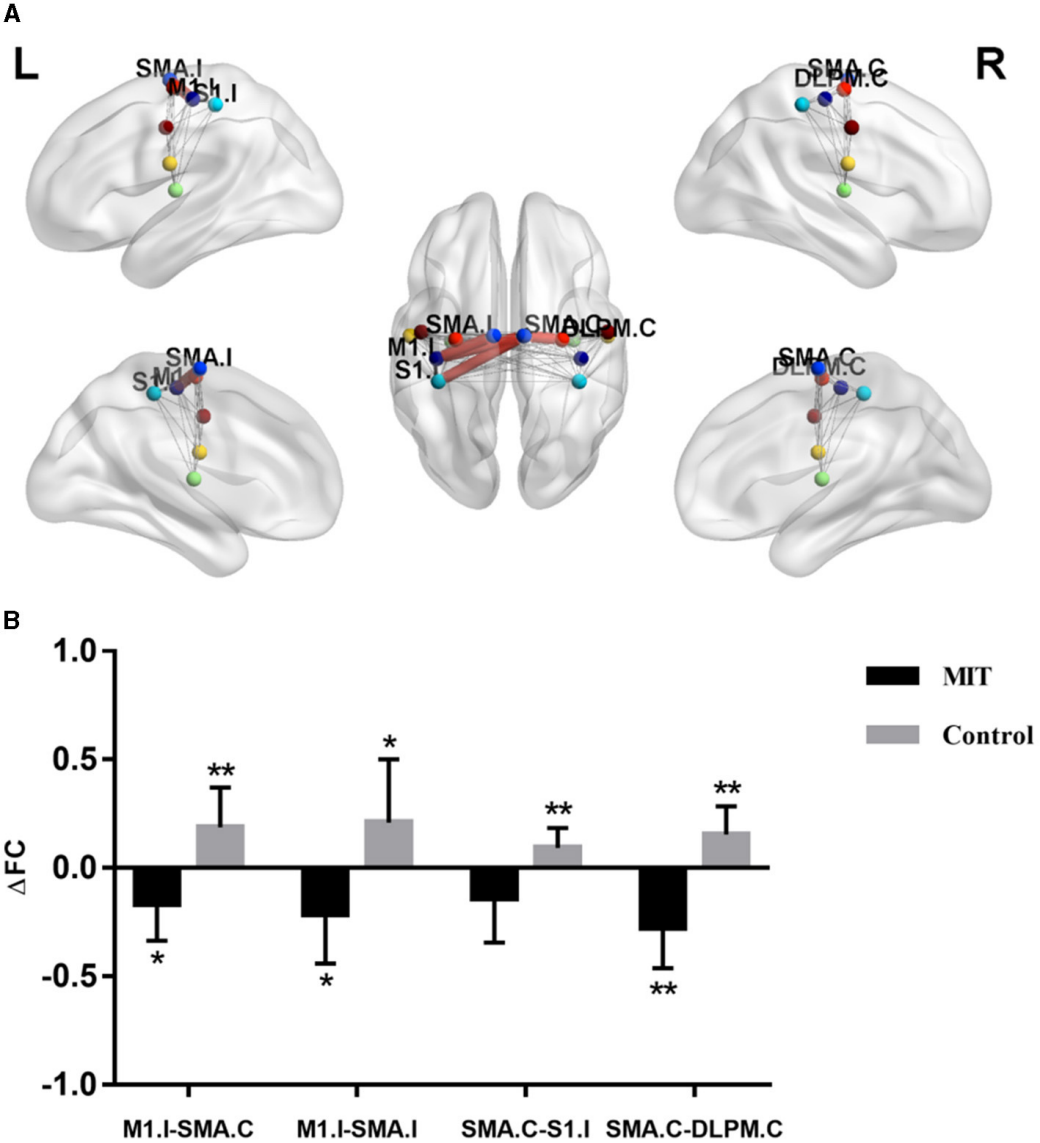
The difference of connectivity within the Sensorimotor Network between the two groups. **(A)** Red thick lines represent functional connectivity (FC) with significant time × group interactions in repeated analysis of covariance. **(B)** Averaged FC change (ΔFC) extracted with significant time × group interactions. Significant changes after intervention in each group were shown as ^*^P < 0.05, ^**^P < 0.01. M1.I, ipsilesional primary motor cortex; SMA.C, contralateral supplementary motor area; SMA.I, ipsilesional supplementary motor area; S1.I, ipsilesional primary somatosensory cortex; DLPM.C, contralateral dorsolateral premotor cortex.

The repeated ANCOVA on ROI-wise connectivity showed significant time × group interactions in the FC between ipsilesional M1 (M1.I) and contralateral SMA (SMA.C), M1.I and ipsilesional SMA (SMA.I), SMA.C and ipsilesional primary somatosensory cortex (S1.I), and SMA.C and contralateral dorsolateral premotor cortex (DLPM.C) (Figure 1A and Supplementary Table S2). *Post hoc* analysis showed that after intervention, the FCs between M1.I and SMA.C, M1.I and SMA.I, and SMA.C and DLPM.C significantly increased in the control group but decreased in the MIT group; while the FC between SMA.C and S1.C significantly increased in the control group but there was no significant difference in the MIT group (Figure 1B). These FCs did not show significant between-group differences at the baseline.

### 3.3 Seed-based whole-brain FC

**Figure 2:**
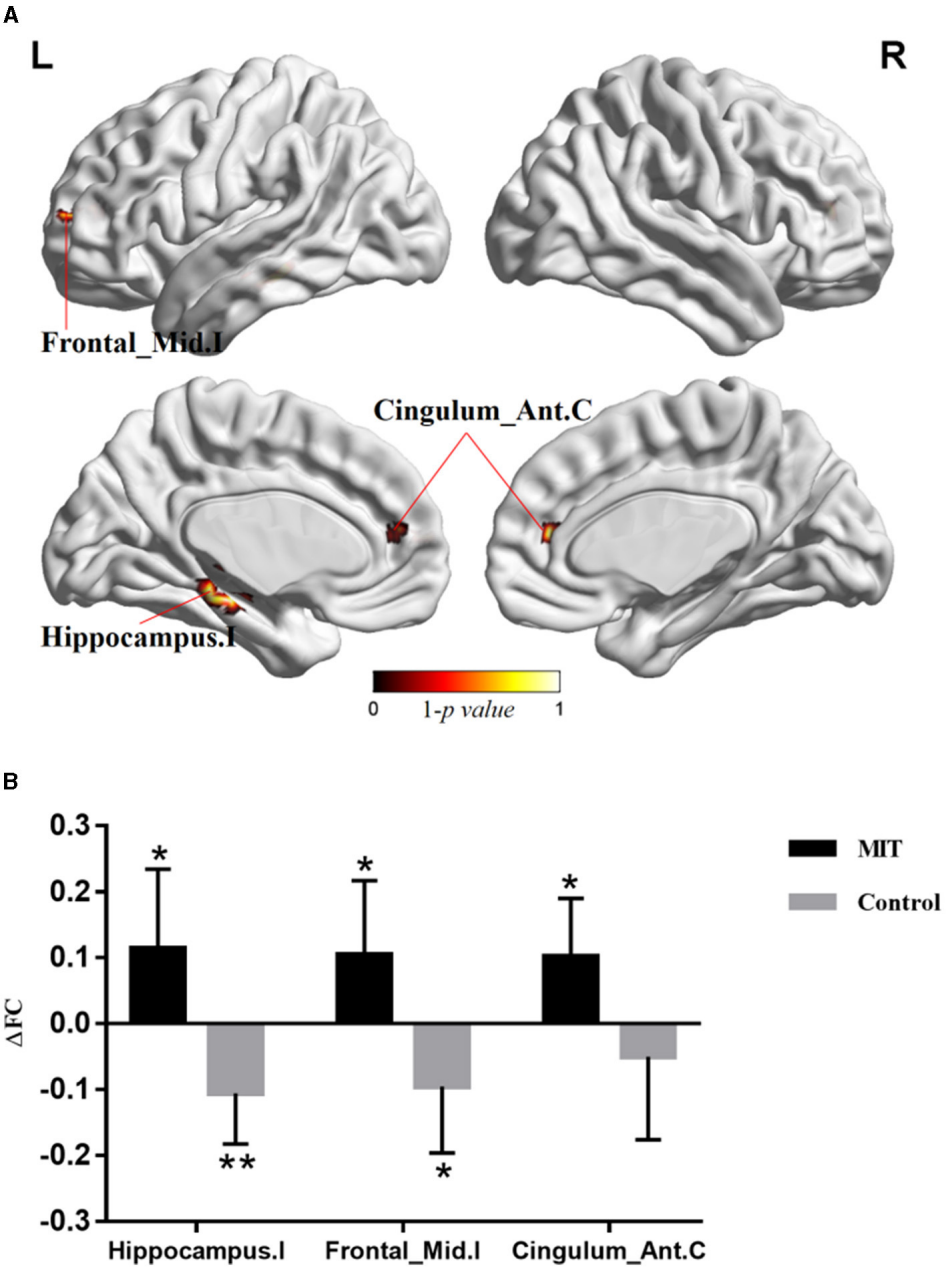
The difference of whole brain functional connectivity (FC) between the two group. **(A)** Brain regions with significant time × group interactions in repeated analysis of covariance. **(B)** Averaged FC change (ΔFC) extracted with significant time × group interactions. Significant changes after intervention in each group were shown as *P < 0.05, **P < 0.01. Hippocampus.I, ipsilesional hippocampal gyrus; Frontal_Mid.I, ipsilesional middle frontal gyrus; Cingulum_Ant.C, contralateral anterior cingulate cortex.

The repeated ANCOVA on seed-based analysis showed significant time × group interactions in the FCs between M1.I and ipsilesional hippocampal gyrus, ipsilesional middle frontal gyrus, and contralateral anterior cingulate cortex (Figure 2A and Table 2). *Post hoc* analysis showed that after intervention, the FCs in ipsilesional hippocampal gyrus and ipsilesional middle frontal gyrus significantly decreased in the control group but increased in the MIT group; while the FC in contralateral anterior cingulate cortex significantly increased in the MIT group but there was no significant difference in the control group (Figure 2B). These FCs did not show significant group differences at the baseline.

**Table 2:** Brain regions with significant time × group interactions in repeated analysis of covariance.

**Region (aal)**	**Peak MNI coordinate**			***P***	**Cluster number**
	**x**	**y**	**z**		
Hippocampus.I	−27	−24	−15	0.007	51
Frontal_Mid.I	−27	57	12	0.037	22
Cingulum_Ant.C	15	42	15	0.024	10

Hippocampus.I, ipsilesional hippocampal gyrus; Frontal_Mid.I, ipsilesional middle frontal gyrus; Cingulum_Ant.C, contralateral anterior cingulate cortex.

### 3.4 Correlations between FC and clinical characteristics

**Figure 3:**
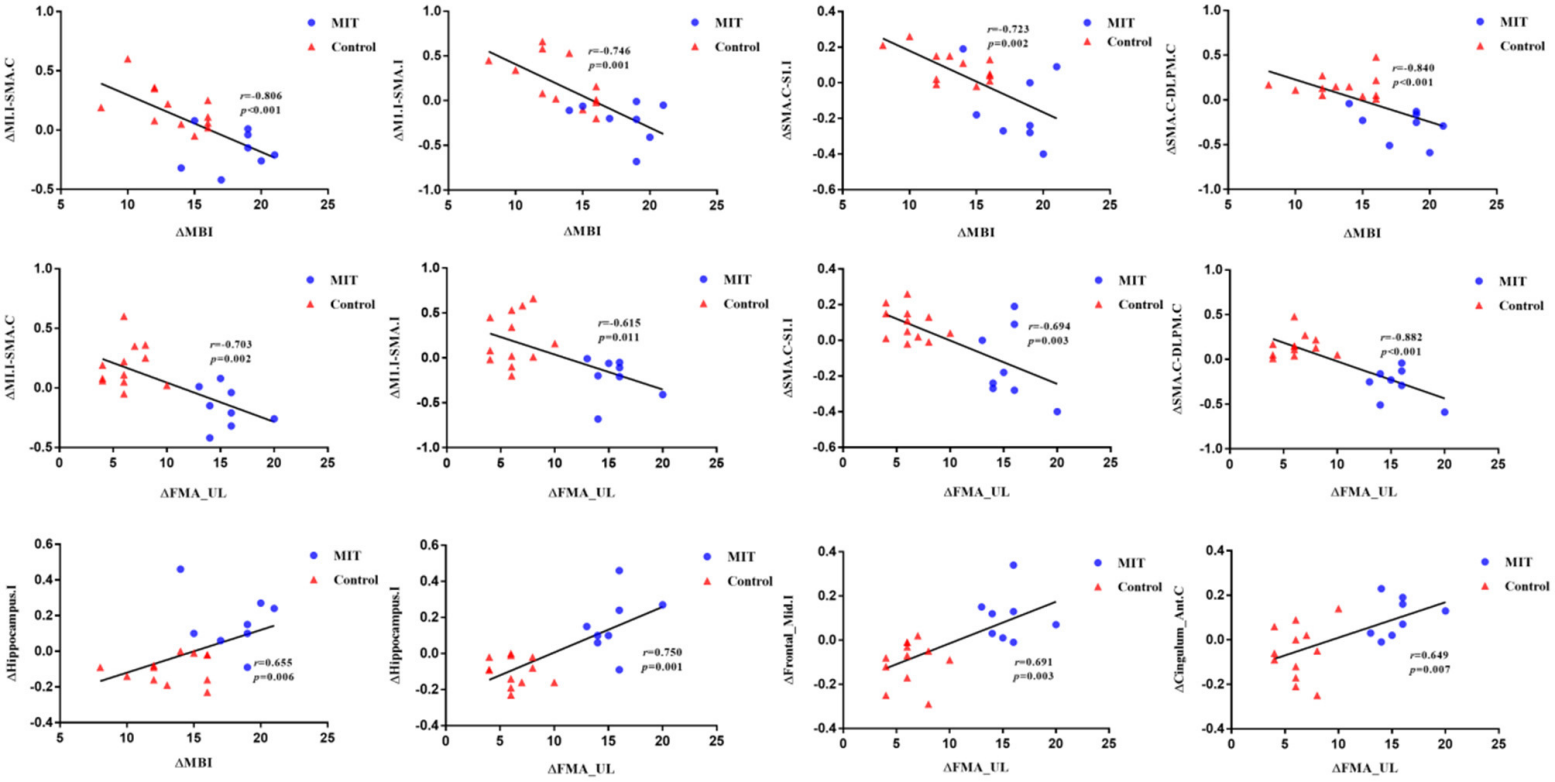
Scatter plots depicting the correlations between ΔFC and ΔMBI or ΔFMA_UL (p < 0.05). Partial correlation coefficients (r) were corrected for age, gender, months after stroke onset, and lesion volume. Scatter plots is fitted with regression line (black line). M1.I, ipsilesional primary motor cortex; SMA.C, contralateral supplementary motor area; SMA.I, ipsilesional supplementary motor area; S1.I, ipsilesional primary somatosensory cortex; DLPM.C, contralateral dorsolateral premotor cortex. Hippocampus.I, ipsilesional hippocampal gyrus; Frontal_Mid.I, ipsilesional middle frontal gyrus; Cingulum_Ant.C, contralateral anterior cingulate cortex; MIT, motor imagery training; MBI, Modified Barthel Index; FMA_UL, Fugl-Meyer Assessment Upper Limb subscale.

For ROI-wise connectivity, as shown in Figure 3, partial correlation analysis revealed that the ΔFCs between the M1.I and SMA.C, M1.I and SMA.I, SMA.C and S1.I, and SMA.C and DLPM.C negatively correlated with the ΔMBI and ΔFMA_UL (Bonferroni-corrected, *P* < 0.05). Also, for seed-based FC, partial correlation analysis showed that the extracted ΔFC of the M1.I seed and the ipsilesional hippocampal gyrus positively correlated with the ΔMBI and ΔFMA_UL (Bonferroni-corrected, *P* < 0.05), and ΔFC of the M1.I seed and ipsilesional middle frontal gyrus and contralateral anterior cingulate cortex positively correlated with ΔFMA_UL (Bonferroni-corrected, *P* < 0.05).

## 4 Discussion

In the current study, we used rs-fMRI to explore the mechanism of the reorganization of the intra-FCs within the SMN and inter-FC between the SMN and other brain regions in stroke patients with hemiplegia who were administered MIT and CRT, and the relationship between the changed FCs and the improvement in upper limb motor function. The results revealed that stroke patients with CRT exhibited increased intra-FCs within the SMN while showing decreased inter-FCs between the SMN and other brain regions. However, in patients with both MIT and CRT, there was a decrease in intra-FCs within the SMN along with an increase in inter-FCs between the SMN and other brain regions. Furthermore, the differences in abnormal intra-FCs within the SMN negatively correlated with the differences in FMA and MBI, and the difference in abnormal inter-FCs of the SMN positively correlated with the differences in FMA and MBI. The results suggested that MIT combined with CRT and CRT alone employ different neural networks.

Firstly, the important finding from the current study is that MIT combined with CRT was superior to CRT alone in improving upper limb function and daily activities in stroke patients with hemiplegia. A large number of previous studies have shown that MIT, as a supplementary technology, can improve motor function in stroke patients (Ietswaart et al., 2011; Liu et al., 2014; Kim and Lee, 2015; Souto et al., 2020; Dai et al., 2022; Villa-Berges et al., 2023). A recent review also clarifies that MIT in combination with other treatment appears to be more effective in improving upper extremity activity than the other treatment alone (Villa-Berges et al., 2023), which is consistent with the results of our study.

In addition, the results showed that the FCs between M1.I and SMA.C, M1.I and SMA.I, SMA.C and DLPM.C, and MA.C and S1.C significantly increased, while FCs between M1.I and ipsilesional hippocampal gyrus and ipsilesional middle frontal gyrus decreased in the control group. Our results suggest that CRT may improve the upper limb motor function of stroke patients mainly through the functional reorganization between SMN. Several longitudinal studies have demonstrated that the FC in the SMN first decreases in the early stages after stroke, then increases in the following weeks or months (Xia et al., 2021). A previous animal study showed that in medium stroke animals, FC of sensorimotor cortex increased 21, 49, and 70 days after stroke, as compared to the loss after 3 days. Medium stroke was characterized mainly by subcortical damage with occasional involvement of some ventrolateral cortical tissues (van Meer et al., 2012). From the animal experiment, we can conclude that with the recovery of stroke, the FC of the sensorimotor cortex is naturally increased, which may be a compensation. Because of ethical issues, we could not set up a separate group without any rehabilitation treatment, so we could not compare whether the increase of FC in the sensorimotor cortex in the CRT group was weaker than that in the process of natural recovery. However, correlation analysis showed that the differences in abnormal intra-FCs of the SMN negatively correlated with the differences in FMA and MBI. It can be seen that the stronger the FC compensatory enhancement within the SMN, the worse the improvement of upper limb motor function in stroke patients. Therefore, we speculate that the increase of FC in the sensorimotor cortex of CRT may be weakened compared with that in the process of natural recovery. CRT may improve motor function by reducing compensatory increased FC in the SMN.

We further explored the brain network mechanism of MIT combined with CRT in improving motor function of stroke patients with hemiplegia. The results showed that FCs between M1.I and SMA.C, M1.I and SMA.I, and MA.C and S1.C significantly decreased, while FCs between M1.I and ipsilesional hippocampal gyrus, ipsilesional middle frontal gyrus, and contralateral anterior cingulate cortex increased in the MIT group. Therefore, we speculated that the neural network mechanism of MIT combined with CRT is different from that of CRT alone. MIT may mainly increase the interaction between SMN and other brain networks.

The hippocampus is involved in procedural memory, a type of memory necessary for motor sequence learning (MSL) (Dolfen et al., 2021). Various models of MSL indicate that the encoding of a new motor skill relies on “cortico-cerebellar, corticostriatal, and cortico-hippocampal” circuits (Doyon et al., 2009; Penhune and Steele, 2012; Albouy et al., 2013). The recovery of motor function is also a process of motor learning; therefore, the increase in connectivity between the SMN and hippocampus is beneficial to the recovery of motor function, which is consistent with our results. Moreover, the hippocampus is implicated in sensorimotor integration (Ekstrom and Watrous, 2014; Burman, 2019). A study has shown that somatosensory targeted memory reactivation during postlearning quiet rest can enhance motor performance via the modulation of hippocampo-cortical responses (Veldman et al., 2023). Meanwhile, some animal studies showed hippocampal theta activity associated with motor behavior (Wyble et al., 2004; Bland et al., 2006; Shin, 2011). Therefore, we hypothesized that the hippocampus improves motor function not only by enhancing motor learning, but also by directly participating in sensorimotor activity, which is analogous to MIT in the current study. MIT is not only a sensorimotor integration process, but also a cognitive training.

The cingulate cortex is a major part of the anatomy of the limbic system. According to the classic, it involves emotions. In view of clinical and experimental findings, the cingulate cortex is involved not only in emotional processes, but also in sensory, motor, and cognitive processes (Vogt et al., 1992). A stereotactic electrical stimulation study showed that the anterior cingulate gyrus controls most complex motor behaviors. The stimulation of the dorsal branch of the cingulate sulcus can induce body orientation, while the hand movement is induced by the stimulation of the middle position of the cingulate gyrus (Caruana et al., 2018). Thus, the anterior cingulate gyrus plays an important role in both cognition and movement, which is consistent with our findings that motor imagery can improve upper limb function.

Similarly, the middle frontal gyrus also plays an important role in cognitive functions, especially in executive functions. A previous study showed that the middle frontal gyrus to be active was registered between 900 and 200 ms prior to the onset of the movement. SMA, premotor cortex, and motor cortex were subsequently activated (Pedersen et al., 1998). This shows that the middle frontal gyrus is also crucial in the movement plan and the initial stage of movement.

Hippocampal gyrus, middle frontal gyrus, and anterior cingulate cortex are related to motor function, but they also play an important role in cognitive function. It can be speculated that MIT mainly improves upper limb motor function through the interaction of cognitive network and the SMN. Similarly, a study has shown that coordinated efforts during bimanual and perhaps other modes of behavior are mediated by regions contributing to higher order functions, which form an interface between cognition and action (Wenderoth et al., 2005). Furthermore, the difference in abnormal FCs in the SMN negatively correlated with the differences in FMA and MBI, and the difference in abnormal FCs between SMN and ipsilesional hippocampal gyrus, ipsilesional middle frontal gyrus, and contralateral anterior cingulate cortex positively correlated with the differences in FMA and MBI. This further indicated that the stronger the FC between the SMN and cognitive network, the more obvious the improvement in upper limb motor function.

## 5 Limitations

Despite the results, there are several limitations to our study. Firstly, we only studied the changes in FC of the SMN before and after treatment in the two groups. We plan to further study the changes in FC of other networks or other characteristics of the networks. Secondly, the sample size was relatively small. According to the latest study, each group may require 17 patients (Wang et al., 2022), but we conducted strict statistics and multiple comparison correction, so our results are reliable. And we are working on increasing patient enrollment. We plan to conduct larger sample size studies with long-term follow-ups. Subsequently, the mechanism of functional reorganization in different periods will be dynamically investigated and the effect of MIT on brain functional reorganization in stroke patients will be elucidated.

## 6 Conclusion

Our current study demonstrated that MIT and CRT impact the brain networks differently and create different patterns of FC of the SMN in the stroke patients. Furthermore, the results showed increased intra-FCs within the SMN in patients with CRT. However, MIT combined with CRT increased FCs of the SMN and cognitive network in the patients. This suggested that CRT may improve the upper limb motor function of stroke patients mainly through the functional reorganization between SMN, while MIT may mainly increase the interaction between SMN and other brain networks. Additionally, correlation analyses showed that the difference in abnormal FCs between the SMN and other brain regions positively correlated with the differences in FMA and MBI. This further verified that MIT improves the upper limb motor function of patients through the interaction between the cognitive network and SMN. Our results provide a basis for further exploration of MIT to improve upper limb function in stroke patients and provide new insights into upper limb rehabilitation in stroke patients.

## Data availability statement

The original contributions presented in the study are included in the article/Supplementary material, further inquiries can be directed to the corresponding authors.

## Ethics statement

The studies involving humans were approved by Human Participants Ethics Committee of the Affiliated Brain Hospital of the Nanjing Medical University (Nos.2017-KY038). The studies were conducted in accordance with the local legislation and institutional requirements. The participants provided their written informed consent to participate in this study.

## Author contributions

WL: Writing – original draft. XC: Writing – original draft. JR: Writing – review & editing. JY: Validation, Writing – review & editing. ZL: Validation, Writing – original draft. YW: Data curation, Writing – original draft. LW: Data curation, Writing – original draft. DL: Validation, Writing – original draft. LL: Writing – review & editing. RG: Writing – review & editing.
