# Supplementary material for: Motor imagery therapy improved upper limb motor function in stroke patients with hemiplegia by increasing functional connectivity of sensorimotor and cognitive networks
Source: Front Hum Neurosci. 2024 Feb 19;18:1295859. doi: 10.3389/fnhum.2024.1295859 (PMC10910033; doi:10.3389/fnhum.2024.1295859)
Supplement: Supplementary file 1 [file Data_Sheet_1.docx]

1. Embodied motor imagery therapy

Embodied motor imagery therapy was performed in the same quiet room each time by an experienced physiotherapist, as follows : (1) Preparation before training : the patient's motor function and imaginative abilities were first systematically assessed before training, then motor imagery movements were designed, and instructional phrases and videos of imaginative movements (including basic movements such as hand opening and closing, arm raising, elbow flexion and extension, and goal-directed movements or activities of daily living such as reaching, grasping and lifting household objects, using a brush or comb) were recorded. (2) Before training begins: the patient's position was first adjusted, the therapist explained the movements that require motor imagery and demonstrated them, and then the patient imitates and masters them with the healthy side according to the instructional phrase. (3) Start of training: training started with full body relaxation of patient according to instructions (2min) → sensory perception training (5-10s) → attention training (10s) → watch movement imagery video of movement (5-10s) → patient closed eyes and imagined movement according to instructions (5-10s) → relaxation (10s) → movement repeated 5 times → start new movement. The content of movement imagery is to imagine the spatial position control and motion intention of body movements in different situational environments, and to fully perceive the consistency with specific movements. (4) Ending the exercise: the patient gradually turned his attention to his body, opened his eyes and relaxed his whole body. It is important to note that during the MIT, patients were instructed to focus on themselves to avoid excessive stress or even anxiety. Also, patients were trained through imagination to store normal movement patterns in memory.

1. Image Acquisition for the Patients

MRI scanning was acquired with a Siemens 3.0-T signal scanner (Siemens, Verio, Germany) in Affiliated Brain Hospital of Nanjing Medical University. All patients lay supine with their head fixed by foam pads with a standard birdcage head coil to minimize head movement. Participants were instructed to remain as still as possible, open their eyes remain awake, and not think of anything. High-resolution T1-weighted images were acquired by 3D magnetization-prepared rapid gradient-echo (MPRAGE) sequence (repetition time [TR] =2,300ms; echo time [TE] =2.85ms; flip angle [FA] =9 degrees; matrix =256ⅹ256; field of view [FOV] =256ⅹ256 mm2; slice thickness/gap =1/0.5 mm; 176 slices covered the whole brain) for image registration and functional localization. The imaging took approximately 260 seconds. Functional images were subsequently collected in the same slice orientation with a gradient-recalled echo-planar imaging pulse sequence (TR= 2,000ms; TE =30ms; FA =90degrees; matrix= 64ⅹ64, FOV= 240ⅹ240 mm2; thickness/gap =4.0/0mm;voxel size = 3.8ⅹ3.8ⅹ4 mm3; slice numbers =30). A total of 251 volumes were obtained in this acquisition sequence and each functional resting-state session lasted approximately 500 seconds.

TableS1. Montreal Neurological Institute coordinates of the 14 ROIs of the SMN

| ROIs | X | Y | Z |
| --- | --- | --- | --- |
| left primary motor cortex | -38 | -22 | 56 |
| right primary motor cortex | 38 | -22 | 56 |
| left supplementary motor area | -8 | -10 | 66 |
| right supplementary motor area | 8 | 10 | 66 |
| left primary somatosensory cortex | -37 | -34 | 53 |
| right primary somatosensory cortex | 37 | -34 | 53 |
| left secondary somatosensory cortex | 52 | -10 | 22 |
| right secondary somatosensory cortex | -52 | -10 | 22 |
| left basal ganglia | -30 | -13 | 8 |
| right basal ganglia | 34 | -12 | 8 |
| left dorsolateral premotor cortex | -28 | -12 | 62 |
| right dorsolateral premotor cortex | 28 | -12 | 61 |
| left ventrolateral premotor cortex | -46 | -8 | 41 |
| right ventrolateral premotor cortex | 52 | -8 | 41 |

TableS2. FCs with significant time × group interactions in the repeated ANCOVA

| ROI-ROI FC | F | P |
| --- | --- | --- |
| M1.I- SMA.C | 18.762 | 0.000 |
| M1.I-SMA.I | 11.995 | 0.003 |
| SMA.C -S1.I | 12.019 | 0.003 |
| SMA.C -DLPM.C | 37.220 | 0.000 |

Abbreviation: FC, functional connectivity; ANCOVA, analysis of covariance; ROI, region of interest; M1.I, ipsilesional primary motor cortex; SMA.C, contralateral supplementary motor area; SMA.I, ipsilesional supplementary motor area; S1.I, ipsilesional primary somatosensory cortex; DLPM.C, contralateral dorsolateral premotor cortex.
